# Phase I trial of CYT997, a novel cytotoxic and vascular-disrupting agent

**DOI:** 10.1038/sj.bjc.6605841

**Published:** 2010-08-24

**Authors:** J D Lickliter, A B Francesconi, G Smith, M Burge, A Coulthard, S Rose, M Griffin, R Milne, J McCarron, T Yeadon, A Wilks, A Cubitt, D K Wyld, P A Vasey

**Affiliations:** 1 Department of Medical Oncology, Royal Brisbane and Women's Hospital, Herston 4029, Queensland, Australia; 2 Leukaemia Foundation Laboratory, Queensland Institute of Medical Research, Herston 4029, Queensland, Australia; 3 Phase I Cancer Trials Program, Monash Medical Centre and Monash Institute of Medical Research, 27-31 Wright Street, Clayton 3168, Victoria, Australia; 4 Cytopia Research Pty Ltd., Richmond 3121, Victoria, Australia; 5 Centre for Magnetic Resonance, University of Queensland, St Lucia 4072, Queensland, Australia; 6 Sansom Institute, University of South Australia, Adelaide 5000, South Australia

**Keywords:** vascular-disrupting agents, CYT997, phase I clinical trial, pharmacokinetics, DCE-MRI

## Abstract

**Background:**

CYT997 is a novel microtubule inhibitor and vascular-disrupting agent with marked preclinical anti-tumour activity.

**Methods:**

This phase I dose-escalation study assessed the safety, tolerability, pharmacokinetics and pharmacodynamics of CYT997 administered by continuous intravenous infusion over 24 h every 3 weeks to patients with advanced solid tumours.

**Results:**

Thirty-one patients received CYT997 over 12 dose levels (7–358 mg m^−2^). Doses up to 202 mg m^−2^ were well tolerated. Dose-limiting toxicities were observed at 269 and 358 mg m^−2^, consisting of grade 3 prolonged corrected QT interval in two patients and grade 3 hypoxia and grade 4 dyspnea in one patient. All toxicities were reversible. The pharmacokinetics of CYT997 were linear over the entire dose range. Dynamic contrast-enhanced magnetic resonance imaging scans showed significant changes in tumour *K*^trans^ values consistent with vascular disruption in 7 out of 11 evaluable patients treated at CYT997 doses of ⩾65 mg m^−2^. Moreover, plasma levels of von Willebrand factor and caspase-cleaved cytokeratin-18 increased post-treatment at higher dose levels. Among 22 patients evaluable for response, 18 achieved stable disease for >2 cycles.

**Conclusions:**

CYT997 was well tolerated at doses that were associated with pharmacodynamic evidence of vascular disruption in tumours.

The ability of solid tumours to promote a pathological neovasculature is essential to their survival, growth and metastasis. Therefore, agents that damage or inhibit the formation of tumour blood vessels have the potential for significant anti-cancer activity (Folkman, 2002). It is critical that these interventions selectively target tumour blood vessels so that vascular toxicity to normal tissues is limited. Encouragingly, there are major biological differences between the immature disorganised microvasculature of malignant tumours and normal microvessel networks, and these differences provide the basis for therapeutic selectivity (Hinnen and Eskens, 2007).

One class of vascular-targeting anti-cancer agents is the vascular-disrupting agents (VDAs). These drugs selectively disrupt endothelial cells within the tumour microvasculature, resulting in rapid shutdown of tumour blood flow (Hinnen and Eskens, 2007; Tozer *et al*, 2008). In animal models, this typically results in necrosis of the central region of the tumour, with a thin peripheral rim of surviving tumour cells that are presumably supplied by vessels in the adjacent normal tissue. Agents in this class include combretastatin A4 phosphate, 5,6-dimethylxanthenone-4-acetic acid, ZD6126 and others (Rustin *et al*, 2003; Stevenson *et al*, 2003; Beerepoot *et al*, 2006; McKeage *et al*, 2006). Although different mechanisms of action are operative, some VDA are tubulin-interactive small molecules that selectively inhibit microtubule polymerisation in endothelial cells (Tozer *et al*, 2008; Schwartz, 2009). Tumour endothelium is dependent on its microtubule cytoskeleton for structural and functional integrity, and disruption of microtubules can trigger a series of changes that shutdown blood flow in the tumour microvasculature (Hinnen and Eskens, 2007). Several VDA are currently in clinical development and some have shown clinical anti-cancer efficacy (Dowlati *et al*, 2002; McKeage *et al*, 2008).

CYT997 is a synthetic small molecule that inhibits tubulin polymerisation, disrupts cellular microtubules and demonstrates potent cytotoxic activity against tumour cell lines *in vitro* (Burns *et al*, 2009b). It also showed significant vascular-disrupting activity in preclinical tumour models (Burns *et al*, 2009a). CYT997 is orally bioavailable and repeat-dose animal toxicology studies have evaluated both intravenous (i.v.) and oral schedules. Common toxicities included hypocellularity of spleen, thymus and bone marrow, leucopenia and mucosal hemorrhage and ulceration in the gastrointestinal tract. Mild bradycardia was observed at higher doses, but there were no other cardiovascular or neurological toxicities (Cytopia Research Pty Ltd, 2009).

We now report the results of a phase I dose-finding study of CYT997 administered by continuous 24-h i.v. infusion in patients with refractory advanced cancer. Pharmacodynamic (PD) evaluations of vascular-disrupting activity were performed, including measurement of plasma von Willebrand factor (vWF) and circulating endothelial cells (CECs), and assessment of the tumour microvasculature with dynamic contrast-enhanced magnetic resonance imaging (DCE-MRI). We also evaluated plasma levels of caspase-cleaved cytokeratin-18 (CK-18) as a surrogate marker of tumour apoptosis.

## Patients and methods

Patients were recruited and followed up at Royal Brisbane and Women's Hospital, and CYT997 infusions were administered in the Q-Pharm phase I unit (Q-Pharm Pty Ltd, Herston, Queensland, Australia). The study was approved by the human research ethics committees of both institutions. All patients gave written informed consent.

### Patient eligibility

Eligible patients were at least 18 years old with a histologically confirmed solid malignant tumour that was refractory to standard treatment or had no available standard therapy. An Eastern Cooperative Oncology Group performance status of ⩽2 and a life expectancy of >3 months were required. Adequate bone marrow, renal and hepatic function for study entry was defined as absolute neutrophil count ⩾1.5 × 10^9^ per litre, platelet count ⩾100 × 10^9^ per litre, creatinine ⩽1.5 × upper limit of normal, total bilirubin ⩽1.5 × upper limit of normal and aspartate aminotransferase and alanine aminotransferase ⩽3 × upper limit of normal (⩽5 × upper limit of normal if liver metastases). A normal left ventricular ejection fraction on a gated heart pool scan or echocardiogram was required. Patients had received no anti-cancer chemotherapy or hormonal therapy in the past 4 weeks (6 weeks if the last regimen included mitomycin-C or nitrosoureas). Female patients of child-bearing potential were required to have a negative serum pregnancy test.

Patients were excluded from the study if they had a history of myocardial infarction or stroke within the past 6 months, unstable angina pectoris or acute ischaemic changes on an electrocardiogram (ECG), a history of diabetic retinopathy, symptomatic peripheral arterial disease or major surgery in the past 30 days. Patients with uncontrolled diarrhea despite optimal medication or any history of acute gastrointestinal bleeding were also excluded. Other exclusion criteria were coexisting illness likely to interfere with trial procedures, known brain metastases and known human immunodeficiency virus infection.

### CYT997 administration

CYT997 was supplied by Cytopia Research in vials containing 100 mg of study drug. On the day of dosing, CYT997 was dissolved in sterile normal saline (50 ml per vial) and filtered twice through a 0.22-*μ*m polyethersulfone filter (Millex GP, Millipore, Billerica, MA, USA). The required dose was added to a 500-ml bag of sterile normal saline and administered by continuous i.v. infusion over 24 h. Doses were repeated every 21 days. Because of grade 2 i.v.-site reactions involving peripheral cannula sites at 23 and 35 mg m^−2^, higher doses were administered via a central venous access device.

### Dose escalation

The starting dose of CYT997 was 7 mg m^−2^, which was approximately one tenth of the severely toxic dose in rats. Doses were escalated according to a modified Fibonacci series. Initially, three patients were entered per dose level (standard dose-escalation phase). As no drug-related toxicity of >grade 2 had been observed by the completion of dose-level 6 (65 mg m^−2^), an accelerated dose-escalation scheme was adopted (Simon *et al*, 1997). This required enrolment of one patient per dose level until the occurrence of either dose-limiting toxicity (DLT) (see below) in one patient or first-cycle grade 2 drug-related toxicity in two or more patients. At that point, standard dose escalation was to be resumed.

Toxicities were graded according to the Common Terminology Criteria for Adverse Events of the National Cancer Institute (version 3.0). Dose-limiting toxicity was based on toxicities during the first cycle of CYT997 and was defined as grade 4 neutropenia lasting ⩾5 days, associated with fever (⩾38.5°C) or requiring antibiotics; grade 4 thrombocytopenia (grade 3 if bleeding); or drug-related non-haematological toxicity ⩾grade 3 (excluding nausea, vomiting and diarrhoea, unless receiving optimal supportive care). In the absence of DLT, escalation proceeded to the next dose level. However, if a DLT was observed, then the dose level was expanded to a planned total of 6 patients. The maximum tolerated dose (MTD) was defined as the dose that resulted in ⩾2 out of 6 patients developing a DLT. Intrapatient dose escalation was permitted in both the standard and accelerated escalation parts of the study, but required that the dose level immediately above had been completed without the occurrence of DLT.

### Patient assessments

Pretreatment evaluation included a medical history and physical examination, assessment of performance status, 12-lead ECG, urinalysis and laboratory studies (including full blood count, coagulation profile, electrolytes, urea, creatinine, liver function tests, glucose, uric acid, calcium and phosphate). Patients were admitted to the Q-Pharm inpatient facility for 48 h with their first CYT997 infusion and for 24 h with subsequent doses. Continuous cardiac monitoring by telemetry, continuous pulse oximetry and frequent vital sign assessments were performed during admissions. For the first CYT997 cycle, laboratory studies and a 12-lead ECG were repeated at 8, 24 and 48 h from starting the infusion and then weekly. For subsequent cycles, laboratory studies and an ECG were performed prior to and at completion of the CYT997 infusion, and laboratory studies were performed weekly. With the demonstration of CYT997-induced prolongation of the corrected QT (QTc) interval at higher doses, the frequency of 12-lead ECG tracings was increased to every 6 h during the first infusion and until 12 h after its completion. Assessment of left ventricular ejection fraction by gated heart pool scan or echocardiogram, and pulmonary function by spirometry and measurement of lung volumes and diffusing capacity, was performed at baseline and after every 2 cycles of CYT997. Radiological evaluation of tumours was performed at baseline and then after every 2 cycles. Efficacy in patients with measurable disease was assessed using Response Evaluation Criteria in Solid Tumors (RECIST) (Eisenhauer *et al*, 2009). Patients were considered evaluable for response if they had at least one measurable tumour lesion at baseline that was reevaluated with the same imaging modality after 2 cycles of study treatment.

### Pharmacokinetic studies

Concentrations of CYT997 in plasma and urine were determined for the first dose of study drug in each patient. Blood samples were drawn immediately prior to starting the infusion and then at 4, 8, 12, 16, 20 and 24 h from the start of infusion, and at 10, 20 and 40 min and 1, 1.5, 2, 4, 6, 8, 12, 24 and 48 h after completion of the infusion. Each sample consisted of ∼5 ml of blood collected in an EDTA-coated tube. Samples were centrifuged at 1300 **g** for 10 min at room temperature within 30 min of collection. Plasma was then transferred to a fresh tube and stored at −80°C, pending analysis. Urine was collected for a 24-h period prior to starting the first CYT997 infusion and a second 24-h period beginning with infusion commencement. Urine volumes were measured and an aliquot was stored at −80°C for analysis.

Analysis of plasma and urine was conducted using validated high performance liquid chromatography–mass spectrometry methods. The area under the concentration of CYT997 in plasma *vs* time curve from the start of the infusion until the last quantifiable concentration (AUC_0−*t*_) was calculated by the linear trapezoidal method using WinNonlin (Version 5.1, Pharsight Corporation, Mountain View, CA, USA). The area beyond the last quantifiable concentration to infinity (AUC_*t*−∞_) was calculated by extrapolation using the terminal rate constant (*K*_el_); the latter calculated from at least three data points of the terminal phase. The two areas were summed to give AUC_o−∞_. Terminal half-life (*t*_½_) was the quotient of 0.693 and *K*_el_. Clearance was calculated as the quotient of the entire i.v. dose infused and AUC_0−*t*_ (CL) and as the quotient of the rate of infusion and the mean of the concentrations at 16 and 20 h (CL_ss_) when steady state was assumed.

### Pharmacodynamic studies

#### Plasma vWF antigen

Blood samples in citrate tubes were centrifuged twice and the plasma was stored at −70°C for assay in batches. The vWF antigen levels in the plasma were determined with the vWF:Ag LIATest immunoturbidometric assay, according to the manufacturer's instructions (Diagnostica-Stago, Asnières-sur-Seine, France). Samples were read using a Stago coagulation analyser. The vWF levels were obtained at baseline and again at 8, 24 and 48 h after the start of the first CYT997 infusion.

#### Circulating endothelial cells

Heparinised peripheral blood samples were centrifuged at 200 **g** for 10 min at room temperature, and the plasma was removed and stored at −70°C for the M30 Apoptosense ELISA assay (see below). The remaining blood cells were resuspended in RPMI 1640, layered over Ficoll-Paque PLUS (GE Healthcare, Little Chalfont, Buckinghamshire, UK) and centrifuged at 970 **g** for 10 min at room temperature with the brake off. The mononuclear cell layer was then collected and washed three times in PBS, prior to staining with monoclonal mouse anti-human CD146-PE, CD31-FITC and CD45-APC antibodies (BD Pharmingen, San Diego, CA, USA). Non-viable cells were stained with 7-amino-actinomycin D (7-AAD, BD Pharmingen). A Becton Dickinson (San Jose, CA, USA) FACSCalibur flow cytometer was used to enumerate CEC as CD146+/CD31+/CD45− cells, as previously described (Bertolini *et al*, 2006). Assays were performed at baseline and again at 24 and 48 h, and 6 days, from starting the first CYT997 infusion.

#### Plasma CK-18 fragment assay

The M30 Apoptosense ELISA assay (Peviva AB, Bromma, Sweden) was used according to the manufacturer's instructions to assay for caspase-cleaved CK-18 fragments in plasma samples isolated during the CEC assay (see above). Each sample was assayed in duplicate. Absorbance was read at 450 nm by a Versamax tunable microplate reader (Molecular Devices, Sunnyvale, CA, USA). The CK-18 fragment assays were performed at baseline and again at 24 and 48 h, and 6 days, from starting the first CYT997 infusion.

#### DCE-MRI scans

Two baseline DCE-MRI scans were performed in the week prior to commencing study treatment to evaluate reproducibility of permeability measures and post-treatment scans were performed at 26 h and 6 days from starting the first CYT997 infusion. Imaging was performed on a 1.5-T Siemens Avanto scanner (Erlangen, Germany). After acquisition of a number of anatomical T2-weighted HASTE images for tumour localisation, four 3D fast gradient echo images were acquired to enable calculation of a baseline T1 map for DCE analysis. These images were acquired using variable flip angles (5°, 10°, 20° and 25°) and a field of view of 220 mm, slice thickness 4 mm, acquisition matrix 128 × 128, repetition time 4.3 ms and four averages in an axial plane. This was followed by a DCE acquisition series at a flip angle of 20°, consisting of 75 scans with a temporal spacing of ∼3 s. Gadolinium-based contrast agent (Gd-DTPA-BMA; Omniscan, Amersham Health AS, Oslo, Norway) was injected as a bolus over 4 s at a dose of 0.1 mmol kg^−1^ of body weight. A vascular input function was measured within a large artery close to the site of the tumour. A similar anatomical location was used for all data in the time series. Permeability maps (*K*^trans^) were generated using the pharmacokinetic modelling method reported by Li *et al* (2000). All follow-up anatomical and permeability maps were carefully registered to the baseline anatomical image acquired at the first imaging time point using an affine transformation. To identify regions of interest (ROI) on permeability maps for statistical analyses of tumour *K*^trans^ values, the first image in the DCE series, acquired without contrast agent, was subtracted from the last image in the series. This approach enabled delineation of the tumour margins exhibiting post-contrast enhancement. This procedure was repeated for both DCE data sets acquired prior to CYT997 treatment and an averaged ROI generated. The averaged ROI was then transferred to the registered permeability maps acquired before and after CYT997 treatment for analysis of *K*^trans^ changes. Results were reported as whole-tumour median *K*^trans^ values, colour-coded *K*^trans^ maps and histogram analyses (Watson *et al*, 2006). Tumour *K*^trans^ maps from all time points were superimposed on the anatomical images acquired during the first baseline DCE-MRI scan, as these were acquired at the highest resolution. For histogram analyses, voxels were grouped in deciles, with the first decile containing the lowest 10% of *K*^trans^ values in the initial baseline scan, the second decile containing the next lowest 10% and so on. Considering the voxels in each decile individually, the differences in *K*^trans^ between the first baseline scan and each of the second baseline, first post-treatment and second post-treatment scans were calculated. Histograms were then constructed for each post-treatment time point so that bars indicated the mean change in *K*^trans^ for each decile of voxels.

#### Statistics

Differences between higher-dose and lower-dose groups with respect to plasma levels of vWF antigen were assessed for statistical significance using an unpaired two-tailed Student's *t*-test. For the histogram analyses of DCE-MRI results, the statistical significance of post-treatment changes in *K*^trans^ in the voxels of a particular decile was determined by comparison with the changes in that decile between the first and second baseline scans. This was achieved using an unpaired Wilcoxon rank-sum test.

## Results

### Patient characteristics

Thirty-one patients were enrolled in the study between June 2005 and July 2007. The baseline characteristics of these patients are summarised in Table 1.

### Dose escalation and MTD

A total of 98 cycles of CYT997 were administered on study (median 2 cycles per patient, range 1–6) over a total of 12 dose levels (7–358 mg m^−2^). Three patients were enrolled per dose level until completion of dose-level 6 (65 mg m^−2^). As no drug-related toxicities of grade >2 had been observed, an accelerated dose-escalation scheme was substituted and one patient was recruited per dose level up to and including 202 mg m^−2^ (Table 2). The first patient to receive 269 mg m^−2^ experienced a DLT, consisting of grade 3 prolongation of the QTc interval. This dose level was therefore expanded to a total of six patients, without additional DLT being observed. Recruitment to the 358 mg m^−2^ dose level then proceeded and 2 out of 3 patients at this level experienced DLT, consisting of grade 3 prolongation of the QTc interval in one patient and grade 3 hypoxia and grade 4 dyspnea in a second patient. This defined 358 mg m^−2^ as the MTD for CYT997 given by 24-h i.v. infusion every 3 weeks.

### Safety and tolerability

Treatment-related adverse events of ⩾grade 2 are shown in Table 2, with the exception of occurrences of grade 2 peripheral i.v.-site reactions that were observed in one patient at each of CYT997 doses of 23 and 35 mg m^−2^. These reactions involved localised induration and inflammation at peripheral i.v. administration sites, which began within 24 h of completing the CYT997 infusion and subsided over several days. A protocol amendment mandated that subsequent dose levels be administered via a central venous access device and no further treatment-related local venous toxicity was observed.

#### Haematological toxicity

CYT997 had no effect on blood counts in most patients. However, one heavily pretreated patient with ovarian cancer who received 269 mg m^−2^ developed grade 3 neutropenia in cycles 1–3 and lesser grades of neutropenia in cycles 4–6. On each occasion, the neutrophil count reached its nadir and recovered to normal within 24 h of completing the CYT997 infusion. There were no episodes of neutropenic fever. The same patient also developed transient grade 1 thrombocytopenia, grade 2 central abdominal cramps and grade 1 diarrhea with a similar time course to the neutropenia. It may be noteworthy that they had previously received abdominal radiation. Another patient developed grade 3 anaemia after receiving CYT997 at 358 mg m^−2^. No changes in the prothrombin time, activated partial thromboplastin time or plasma fibrinogen level were observed at any dose level.

#### Prolonged QTc interval

One patient at each of CYT997 doses of 269 and 358 mg m^−2^ developed grade 3 prolongation of the QTc interval, with the maximum QTc interval observed reaching 518 ms. The patient treated at 269 mg m^−2^ had a right bundle branch block and grade 1 prolongation of the QTc interval at baseline, whereas the patient treated at 358 mg m^−2^ had a normal baseline QTc interval. In both patients, the QTc-interval prolongation reduced to ⩽grade 1 within 8 h of completing the CYT997 infusion. Grade 2 prolonged QTc interval was seen in a second patient at 358 mg m^−2^, which followed early stopping of the first CYT997 infusion because of dyspnea and hypoxia (see below). No ventricular arrhythmias were associated with prolongation of the QTc interval during this study.

#### Other non-haematological toxicities

Three additional patients developed grade 3–4 non-haematological toxicities. The first of these patients had a pleural mesothelioma and was treated at 269 mg m^−2^. They experienced grade 3 chest pain on day 4 of their third cycle of CYT997. This had the clinical features of tumour pain and took 5 days to decrease in severity to <grade 2. Similar pain of maximum grade 2 severity occurred in the same patient during other cycles. The second patient developed transient uniocular visual loss on day 4 of their second CYT997 cycle, which was dosed at 269 mg m^−2^. This resolved after 1 min without sequelae. Subsequent examination of the optic fundus was normal and a carotid doppler study revealed no significant stenoses. This event did not affect dose escalation because it occurred during the second cycle. However, the first CYT997 infusion in this patient was stopped early after 17 h because of a thrombosis related to their peripherally inserted central catheter, and the second dose was the first given at the allocated dose level. No further doses were given to this patient. The third patient had a history of radiation to a lung mass and received CYT997 at 358 mg m^−2^. They developed grade 3 hypoxia and grade 4 dyspnea during infusion of study drug, which was ceased early after 15 h. The clinical status then significantly improved and the hypoxia resolved over the next several hours. A 12-lead ECG showed sinus tachycardia and no other acute changes. Chest X-ray revealed some increase in a pre-existing unilateral pleural effusion (from mild to moderate) and subtle bilateral interstitial lung opacities that had cleared on a repeat film the next day. Routine haematology and biochemistry studies were unrevealing apart from a transient grade 2 elevation of plasma lactate dehydrogenase. No further CYT997 was given to this patient.

Several grade 2 non-haematological toxicities were observed in one to two patients each, including hypertension, elevated creatinine, proteinuria, nausea, abdominal pain, headache and fever (Table 2). Of note, patient 19 developed grade 2 elevation in plasma creatinine after their dose was escalated from 86 to 114 mg m^−2^. This patient had previously undergone a nephrectomy for renal cell carcinoma and had grade 1 elevated creatinine and microhaematuria at baseline. The plasma creatinine level increased from 119 *μ*mol l^−1^ at baseline to 174 *μ*mol l^−1^ at 8 h into the CYT997 infusion. Study drug infusion was ceased immediately, and the creatinine returned to baseline levels within 2 days. No nephrotoxicity was observed in any other patient. All toxicities were reversible. In contrast to most other microtubule-targeting anti-cancer agents, CYT997 was not associated with the development of peripheral neuropathy.

### Pharmacokinetics

**Figure 1:**
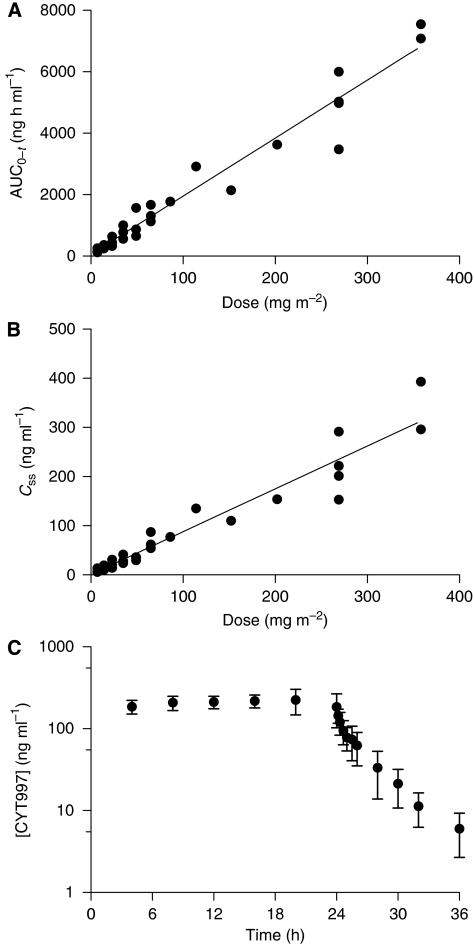
Pharmacokinetic profile of CYT997 given by continuous 24-h i.v. infusion. (**A**) Area under the plasma concentration–time curve (AUC_0−*t*_), and (**B**) plasma concentration at steady state (*C*_ss_), *vs* CYT997 dose level. (**C**) Mean plasma concentration of CYT997 plotted against time from the infusion start for the 269 mg m^−2^ dose level (*n*=4); error bars indicate s.d.

Pharmacokinetic data summarised by dose level are provided in Table 3. For five subjects, it was not possible to calculate a terminal rate constant and, therefore, AUC_0−∞_. However, the extrapolated area from the 23 subjects in which it was possible to calculate the rate constant represented no more than 2.6% of the total area. Therefore, AUC_0−*t*_ was used for the calculation of CL in all subjects. There was a highly significant linear relationship between AUC_0−*t*_ and the total dose of CYT997 infused (*P*<0.001; Figure 1A), indicating that the pharmacokinetics of CYT997 were linear over the entire 50-fold dose range investigated in this study (7–358 mg m^−2^). The plasma concentrations of CYT997 at steady state (*C*_ss_, Table 3) were the maximum concentrations achieved during the infusions. Similar to the AUC_0−*t*_, *C*_ss_ increased in a dose-proportional manner across the entire dose range (Figure 1B). Individual values for CL extended from 0.59 to 1.56 l h^−1^ kg^−1^, with the values for CL calculated from AUC_0−*t*_ being, on average, about 10% less than the values calculated from the concentrations at steady state. A small percentage (<0.7%) of the i.v. dose was excreted unchanged in urine, suggesting extensive hepatic clearance. The values for the apparent volume of distribution (mean 6.5 l kg^−1^; range 2.6–10.6 l kg^−1^) suggest moderate-to-extensive distribution beyond the central compartment. Plasma concentration data indicate a bi-exponential decline in CYT997 levels following cessation of the 24-h infusion (Figure 1C). The mean terminal elimination half-life was 4.4 h (range 1.7–7.5 h).

### Pharmacodynamic studies

#### Plasma vWF levels

**Figure 2:**
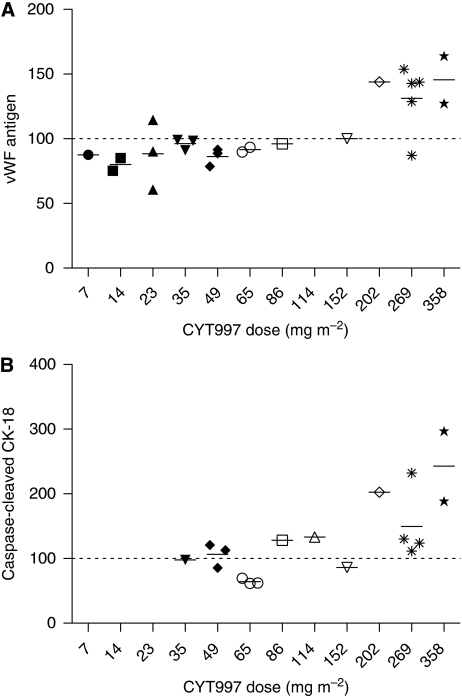
Vertical scatter plots of (**A**) von Willebrand factor (vWF) antigen levels and (**B**) caspase-cleaved cytokeratin-18 (CK-18) levels in plasma at 24 h after commencing the initial CYT997 infusion, grouped by CYT997 dose level. Values are expressed as a percentage of baseline values for each patient and mean values for each dose level are indicated by horizontal bars. For caspase-cleaved CK-18 levels, each data point represents the mean of duplicate assays. Note that there was only one patient per dose level at four of the dose levels (86, 114, 152 and 202 mg m^−2^).

Von Willebrand factor is present in high concentrations in endothelial cells and plasma levels of vWF antigen increase following endothelial cell injury (Lee *et al*, 2005). Plasma vWF antigen was therefore used in the current study as a surrogate marker of endothelial damage because of vascular-disrupting activity. Adequate baseline and post-treatment data were available on 26 patients, but two were excluded due to an unexplained borderline-low vWF antigen level at baseline or the occurrence of a line-related thrombosis during CYT997 infusion. Figure 2A shows that plasma vWF antigen increased at 24 h after commencing CYT997 in patients receiving doses of ⩾202 mg m^−2^. At this time point, the mean (±s.d.) vWF level was 136% (±23%) of baseline for doses ⩾202 mg m^−2^, which was significantly elevated (*P*<0.001) when compared with the mean level of patients receiving <202 mg m^−2^. Levels in the higher-dose group declined to 123% (±25%) of baseline by 48 h, but were still significantly higher (*P*<0.05) than those in lower-dose patients.

#### Circulating endothelial cells

CEC were measured in the peripheral blood of 17 patients as CD146+/CD31+/CD45− cells. The 14 patients with evaluable data received CYT997 at doses ranging from 49 to 358 mg m^−2^. Post-treatment increases in CEC were seen in only one patient (patient 29), who was treated at the highest dose level. In this case, CEC were essentially undetectable at baseline (<0.01% of MNC) and then increased to 0.09% and 0.30% of MNC at 48 h and 6 days, respectively, after commencing CYT997. The CEC detected at these latter time points stained positively with 7-AAD, indicating that they were non-viable.

#### Caspase-dependent CK-18 fragments

Plasma levels of caspase-cleaved CK-18 measured with the M30 ELISA assay have been used to quantify apoptosis induced in epithelial cancers by chemotherapy or other treatments (de Haas *et al*, 2008). Seventeen patients in the current study were evaluable for the analysis of caspase-cleaved CK-18 levels, which were observed to increase at 24 h after commencing CYT997 in a dose-dependent manner (Figure 2B). The majority of patients showing increases had epithelial carcinomas or mesothelioma, which typically express CK-18 (Moch *et al*, 1993). One patient with metastatic vaginal leiomyosarcoma also had a significant post-treatment rise in M30 epitope, although it is notable that this sarcoma subtype expresses CK-18 in 30% of cases (Chu and Weiss, 2002).

#### DCE-MRI

Fifteen patients underwent DCE-MRI scans before and after their first CYT997 infusion and, of these, 11 patients had evaluable DCE-MRI data. Results on four patients were not evaluable because of excessive patient motion during scanning. All evaluable patients received CYT997 doses of ⩾65 mg m^−2^, and therefore 11 out of 16 patients treated at doses from 65 to 358 mg m^−2^ were analysed. Whole-tumour median *K*^trans^ values at baseline and both post-treatment time points are shown in Table 4. The *K*^trans^ values significantly decreased in five patients (18, 20, 21, 25 and 26), which is consistent with a reduction in tumour blood flow in response to study drug in these individuals (Galbraith *et al*, 2003). In two other patients (19 and 30), there was a significant post-treatment increase in tumour *K*^trans^. Maximal response in tumour *K*^trans^ occurred at 26 h after starting the CYT997 infusion in patient 25, whereas in all other cases maximal responses were at 6 days. DCE-MRI results were also subjected to histogram analysis. Post-treatment time points where the majority of histogram deciles revealed a statistically significant change in *K*^trans^ (*P*<0.05 for individual deciles) occurring in the same direction as the change in the whole-tumour median *K*^trans^ value are flagged with bold type in Table 4. Importantly, there is a close match between time points showing substantial changes in whole-tumour median *K*^trans^ and those identified by histogram analysis as revealing statistically significant *K*^trans^ changes.

**Figure 3:**
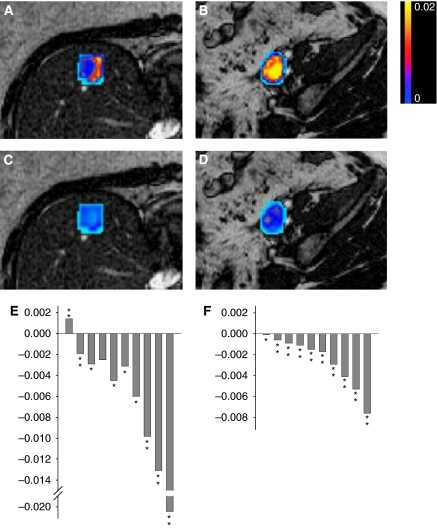
Tumour *K*^trans^ maps derived from DCE-MRI scans of patients 20 (**A**, **C**) and 26 (**B**, **D**). Patient 20 received 114 mg m^−2^ of CYT997 and the lesion shown is a liver metastasis of non-small cell lung cancer. Patient 26 received 269 mg m^−2^ and images show a pelvic nodal metastasis of ovarian carcinoma. Results at baseline (**A**, **B**) and 6 days following CYT997 administration (**C**, **D**) are shown. Maps are colour coded, and the scale (shown at right) extends from a maximum *K*^trans^ of 0.02 min^−1^ (yellow end) to zero (blue end). Histograms indicating the mean change in *K*^trans^ for each decile of voxels at 6 days following CYT997 treatment are also shown for patients 20 and 26 (**E** and **F**, respectively). The bars are arranged so that the decile with the lowest *K*^trans^ values at baseline is on the left of the histogram and the decile with the highest is on the right. Negative values indicate a fall in *K*^trans^ after study drug, compared with baseline. Asterisks indicate statistical significance: ^*^P<0.05, ^**^P<0.0002.

Figure 3 shows *K*^trans^ maps for two of the patients who developed reductions in tumour *K*^trans^ following CYT997 treatment. Images from patient 20 show a liver metastasis of non-small cell lung cancer, with a vascularised rim and presumably necrotic interior at baseline. Six days following CYT997 administration, a major reduction in *K*^trans^ in the tumour rim was observed, consistent with marked reduction in tumour perfusion. Similarly, the *K*^trans^ maps from patient 26 indicate marked and widespread reduction in perfusion of an ovarian carcinoma metastasis at 6 days following CYT997 administration. Histogram analyses were confirmatory in both patients, with statistically significant decreases in tumour *K*^trans^ seen in most or all deciles at 6 days post-treatment (Figure 3E and F). Interestingly, the deciles of voxels with greatest baseline *K*^trans^ values (at right in the histograms) were associated with the greatest fall in *K*^trans^ following CYT997 treatment. A similar pattern was observed in other patients showing significant CYT997-induced changes in tumour *K*^trans^ (data not shown).

**Figure 4:**
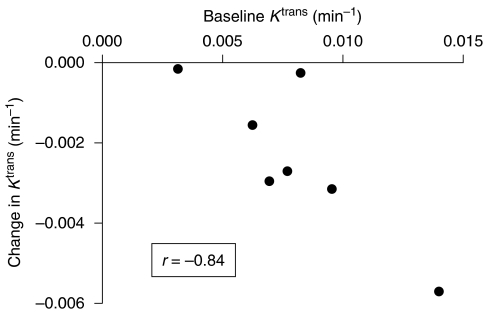
Correlation between the whole-tumour median *K*^trans^ value at baseline and the maximum decrease in whole-tumour median *K*^trans^ observed following CYT997 treatment. Only data from patients with a fall in median *K*^trans^ at one or both post-treatment time points are shown.

As noted above, all patients with evaluable DCE-MRI data received CYT997 doses between 65 and 358 mg m^−2^. Within this dose range, no clear relationship was observed between CYT997 dose level and the likelihood or magnitude of changes in tumour *K*^trans^. Baseline whole-tumour median *K*^trans^ values, however, were correlated (*r*=−0.84) with the extent of *K*^trans^ reduction observed post-treatment (Figure 4). A correlation persisted (*r*=−0.58) when the outlying patient with the highest baseline *K*^trans^ and greatest *K*^trans^ fall was excluded from the analysis.

### Clinical outcomes

Twenty-two patients were evaluable for response. There were no objective responses by RECIST criteria. Stable disease for >2 cycles was achieved in 18 patients (82%), and 6 patients (27%) completed all 6 cycles of CYT997 prescribed by the clinical trial protocol. Most notable were two study participants (patients 21 and 22) who had symptomatic progressive disease prior to study entry. Both remained in ongoing stable disease beyond 6 cycles of study treatment and each received an additional 2 cycles of CYT997 off study, before developing progressive disease. Patient 21 (mesothelioma) received 152 mg m^−2^ in cycles 1 and 2, and 202 mg m^−2^ in cycles 3–8. Patient 22 (tracheal adenocarcinoma) received 202 mg m^−2^ in all cycles. There was no correlation between the duration of stable disease and the degree of reduction in *K*^trans^ following CYT997 administration for the entire group of evaluable patients. However, patient 21 and another patient who achieved stable disease for 6 cycles (patient 26, ovarian carcinoma) both had significant post-treatment falls in *K*^trans^ (Table 4; Figure 3).

## Discussion

We describe results of the first-in-human clinical trial of the cytotoxic and VDA CYT997. As shown in Table 2, CYT997 was well tolerated when given as a 24-h i.v. infusion every 3 weeks at doses up to and including 202 mg m^−2^. Grade 3 and 4 toxicities were observed at higher dose levels, including prolonged QTc interval, transient uniocular visual loss and dyspnea with hypoxia. However, all CYT997 toxicities were reversible without sequelae. The maximum QTc interval observed in the current study was 518 ms, and no ventricular tachyarrhythmias were associated with QTc prolongation in any patient. Dose-related QTc-interval prolongation has been reported with other VDA (Cooney *et al*, 2004; McKeage *et al*, 2006). It is notable that the episode of grade 3–4 dyspnea and hypoxia observed in our study occurred in a patient with a history of thoracic radiation therapy. Furthermore, fatal bowel toxicity was reported in a trial of combretastatin A4 phosphate in a patient with previous abdominal radiation (Rustin *et al*, 2003). It is therefore possible that ionising radiation may sensitise the microvasculature of normal tissues to VDA toxicity. Although the 358 mg m^−2^ dose level was determined as the MTD in our study, the dose level below (269 mg m^−2^) was considered too toxic to be the recommended dose for phase II studies. One patient at 269 mg m^−2^ developed a DLT (QTc-interval prolongation), and another patient at this dose level experienced a grade 4 visual disturbance in cycle 2. The next dose level down (202 mg m^−2^) might have been explored as a potential single-agent phase II dose. However, we decided against this as CYT997 will likely be evaluated further in combination with other anti-cancer drugs, rather than as a single agent. Trials of such combinations will use still lower starting doses of CYT997 and use a limited dose-escalation strategy to establish the recommended dose.

Pharmacokinetic studies revealed that *C*_ss_ (equivalent to *C*_max_) and AUC_0−*t*_ were proportion to CYT997 dose (Table 3; Figure 1). In preclinical investigations, the IC_50_ of CYT997 in diverse cancer cell lines was in the range 10–100 nM (Burns *et al*, 2009a). Doses at and above 65 mg m^−2^ in the current trial achieved plasma concentrations at steady state of >100 nM and for the three dose levels immediately below 269 mg m^−2^ the *C*_ss_ ranged from 253 to 354 nM. Therefore, well-tolerated doses of CYT997 resulted in steady-state plasma levels that were up to 3.5 times higher than the IC_50_ of the most resistant cell line tested and up to 35 times higher than the IC_50_ of the most sensitive cell line.

Von Willebrand factor levels in plasma were assayed as a PD indicator of endothelial cell injury following CYT997 treatment. Levels significantly increased in patients dosed at 202 mg m^−2^ and above (Figure 2A), which is consistent with dose-dependent CYT997-induced vascular disruption and potentially with targeting of tumour vasculature. However, it is noteworthy that all but one of the patients showing a significant rise in plasma vWF received doses ⩾269 mg m^−2^. These dose levels were associated with grade 3–4 cardiovascular toxicities in some patients and therefore injury to normal endothelium is another potential source of the increased plasma vWF. It is not possible to distinguish between these possibilities based on our data. We also assayed CEC as an alternative biomarker of vascular disruption. Only one patient (who was dosed at the MTD) showed an increase. The significance of this finding is uncertain, but CEC levels do not appear to be a useful indicator of CYT997-induced vascular disruption.

Caspase-cleaved CK-18 significantly increased in plasma after CYT997 treatment and the threshold dose for this effect may have been as low as 86 mg m^−2^ (Figure 2B). These results indicate that apoptosis was triggered in CK-18 expressing cells and are consistent with an anti-tumour effect of CYT997. Clearly, however, they could also reflect subclinical toxicity to normal epithelial tissues.

DCE-MRI scans evaluate perfusion and endothelial permeability in tumour microvasculature and therefore complement plasma PD biomarkers by providing both anatomical and physiological information. We observed changes in serial DCE-MRI scans that were consistent with significant CYT997-induced reductions in tumour perfusion in 5 out of 11 evaluable patients (Table 4; Figure 3). Moreover, two additional patients showed a significant increase in tumour *K*^trans^. The biology underlying these latter changes is not fully understood, but could involve increases in microvascular permeability because of lesser degrees of vascular disruption by CYT997 (Seshadri *et al*, 2007). The timing of post-treatment DCE-MRI scans is likely to be critical to an optimal assessment of VDA activity. We performed scans at 26 h and 6 days from starting the CYT997 infusion and therefore even major effects on *K*^trans^ occurring between these time points may have been missed. Nonetheless, the available DCE-MRI observations suggest that CYT997 possesses significant VDA activity.

The threshold dose for induction of vascular disruptive effects by CYT997 has not been defined, as significant *K*^trans^ changes were observed at the lowest dose level for which evaluable DCE-MRI data were available (65 mg m^−2^). In addition, no convincing dose–response relationship was evident as doses increased above this level. CYT997 therefore induced changes consistent with vascular disruption at doses well below its MTD, which has also been reported for other VDA (McKeage *et al*, 2006). The extent to which CYT997 affected tumour microvasculature was, however, related to the median *K*^trans^ of the tumour at baseline (Figure 4). This suggests that tumours with more extensive and/or leaky neovasculature were more susceptible to vascular disruption by this agent. Moreover, areas within an individual tumour that possessed the highest *K*^trans^ values at baseline were subject to the greatest change in blood flow or permeability. CYT997 may therefore be most effective as a VDA against malignancies with an extensive abnormal vasculature. It is notable that combretastatin A4 phosphate also triggered greater changes in *K*^trans^ in tumours with higher baseline *K*^trans^ levels and appeared to have particular activity in thyroid tumours, which are often highly vascular (Dowlati *et al*, 2002; Stevenson *et al*, 2003).

In summary, CYT997 administration was associated with changes in plasma and imaging biomarkers that were consistent with vascular disruption in tumours. These changes were observed in some patients at well-tolerated doses. Our results therefore support the further clinical evaluation of CYT997, which, based on clinical experience with other vascular-targeting agents, might optimally be performed in combination with other anti-cancer therapeutics.

## Figures and Tables

**Table 1:** Patient characteristics

**Characteristic**	**No. of patients**
Total patients	31
	
*Age (years)*	
Median	57
Range	21–75
	
*Sex*	
Male	15
Female	16
	
*ECOG performance status*	
0	8
1	14
2	9
	
*Tumour type*	
Melanoma	5
Mesothelioma	5
Renal cell	4
Breast	2
Prostate	2
Colorectal	2
Head and neck	2
Other	9

Abbreviation: ECOG=Eastern Cooperative Oncology Group.

**Table 2:** Toxicity of CYT997

**Dose level**	**49 mg m^−2^**			**65 mg m^−2^**			**86 mg m^−2^**			**114 mg m^−2^**			**152 mg m^−2^**			**202 mg m^−2^**			**269 mg m^−2^**			**358 mg m^−2^**		
**No. of patients**	**3**			**3**			**1**			**1**			**1**			**1**			**6**			**3**		
**Toxicity grade**	**2**	**3**	**4**	**2**	**3**	**4**	**2**	**3**	**4**	**2**	**3**	**4**	**2**	**3**	**4**	**2**	**3**	**4**	**2**	**3**	**4**	**2**	**3**	**4**
*Haematological*																								
Neutropenia	−	−	−	−	−	−	−	−	−	−	−	−	−	−	−	−	−	−	−	**1**	−	−	−	−
Lymphopenia	−	−	−	−	−	−	−	−	−	−	−	−	−	−	−	−	−	−	−	**1**	−	−	−	−
Anaemia	−	−	−	−	−	−	−	−	−	−	−	−	−	−	−	−	−	−	−	−	−	−	**1**	−
																								
*Non-haematological*																								
Prolonged corrected QT interval	−	−	−	−	−	−	−	−	−	−	−	−	−	−	−	−	−	−	−	1*	−	**1**	1*	−
Hypertension	−	−	−	−	−	−	−	−	−	−	−	−	−	−	−	−	−	−	**1**	−	−	−	−	−
Nausea	−	−	−	−	−	−	−	−	−	−	−	−	−	−	−	**1**	−	−	−	−	−	−	−	−
Pain – abdomen	−	−	−	**1**	−	−	−	−	−	−	−	−	−	−	−	−	−	−	**1**	−	−	−	−	−
Pain – chest/thorax	−	−	−	−	−	−	−	−	−	−	−	−	−	−	−	−	−	−	−	**1**	−	−	−	−
Dyspnea	−	−	−	−	−	−	−	−	−	−	−	−	−	−	−	−	−	−	−	−	−	−	−	1*
Hypoxia	−	−	−	−	−	−	−	−	−	−	−	−	−	−	−	−	−	−	−	−	−	−	1*	−
Headache	−	−	−	**1**	−	−	−	−	−	−	−	−	−	−	−	−	−	−	−	−	−	−	−	−
Visual disturbance	−	−	−	−	−	−	−	−	−	−	−	−	−	−	−	−	−	−	−	−	**1**	−	−	−
Fever	−	−	−	−	−	−	−	−	−	−	−	−	−	−	−	−	−	−	−	−	−	**1**	−	−
Elevated creatinine	−	−	−	−	−	−	−	−	−	**1**	−	−	−	−	−	−	−	−	−	−	−	−	−	−
Proteinuria	−	−	−	−	−	−	−	−	−	−	−	−	−	−	−	−	−	−	−	−	−	**1**	−	−

Number of patients with adverse events of grade ⩾2 that were possibly, probably or definitely related to CYT997 are shown. Several episodes of the same toxicity in the one patient are scored as one event and only the worst grade is shown. Multiple different toxicities occurring simultaneously in the one patient are scored as separate toxicities. The highest eight dose levels are shown (dose levels 5–12). Dose-limiting toxicities are asterisked.

**Table 3:** Pharmacokinetics of CYT997

**Dose (mg m^−2^)**	**No. of patients**	***C*_ss_ (ng ml^−1^)^a^**	**AUC_0−*t*_ (ng h ml^−1^)^a^**	**Terminal *t*_½_ (h)^a^**	**CL (l h^−1^ kg^−1^)^a^**	***V*_app_ (l kg^−1^)^a^**
7	3	8.5 (4.4)	178 (68)	2.8 (0.6)	0.89 (0.23)	3.5 (1.0)
14^b^	3	15.5 (4.9)	312 (58)	6.6 (1.2)	0.93 (0.20)	7.8 (1.4)
23	3	22.0 (8.5)	470 (153)	4.3 (1.2)	1.09 (0.39)	6.3 (0.8)
35^b^	3	31.2 (9.2)	782 (221)	NE	1.04 (0.26)	NE
49^b^	3	33.5 (3.2)	1030 (480)	3.4 (2.4)	1.16 (0.50)	4.1 (0.4)
65	3	67.8 (17.4)	1370 (280)	4.2 (1.1)	1.05 (0.26)	6.1 (0.3)
86	1	77.3	1780	5.4	1.07	8.3
114	1	135	2920	4.6	0.85	5.7
152	1	110	2140	3.9	1.50	8.4
202	1	154	3630	4.5	1.36	8.7
269	5^c^	217 (57)	4570 (1130)	5.1 (1.7)	1.11 (0.23)	8.2 (3.0)
358	2^c^	345 (69)	5640 (2910)	4.6 (2.3)	0.99 (0.18)	6.8 (4.6)

Abbreviations: *C*_ss_=concentration at steady state; AUC_0−*t*_=area under the CYT997 plasma concentration *vs* time curve from the start of the infusion until the last quantifiable concentration; CL=clearance; *V*_app_=apparent volume of distribution; NE, not evaluable.

a Pharmacokinetic parameters represent the mean (s.d.) for the corresponding dose level.

b The concentrations in plasma profile for one subject at 14 mg m^−2^, all three at 35 mg m^−2^ and one at 49 mg m^−2^ could not be extrapolated to infinity and, therefore, the number of subjects available for calculation of mean terminal *t*_½_ was reduced accordingly.

c Data were not available on one patient in each of the 269 and 358 mg m^−2^ dose levels because of early termination of CYT997 infusions.

**Table 4:** DCE-MRI evaluation of tumour vasculature

			**Baseline**		**Post-treatment**	
**Patient no.**	**Tumour type**	**CYT997 dose (mg m^−2^)**	**First**	**Second**	**26 h**	**6 days**
18	Renal cell	65	0.0065	0.0074	0.0062	**0.0040**
19	Renal cell	86	0.0093	0.0093	0.0108	**0.0109**
20	NSCLC	114	0.0133	0.0147	0.0153	**0.0083**
21	Mesothelioma	152	0.0068	0.0057	**0.0062**	**0.0047**
22	Tracheal	202	0.0042	0.0060	0.0065	0.0061
24	Mesothelioma	269	0.0083	0.0082	0.0080	0.0081
25	Prostate	269	0.0101	0.0090	**0.0064**	0.0099
26	Ovarian	269	0.0074	0.0080	0.0088	**0.0050**
27	Breast	269	0.0030	0.0033	0.0034	0.0030
29	Mesothelioma	358	0.0050	0.0054	0.0057	0.0052
30	Leiomyosarcoma	358	0.0073	0.0073	**0.0089**	**0.0094**

Abbreviations: DCE-MRI, dynamic contrast-enhanced magnetic resonance imaging; NSCLC=non-small cell lung cancer.

Whole-tumour median *K*^trans^ values are shown for the first and second baseline DCE-MRI scans and the two post-treatment scans (26 h and 6 days after starting the first CYT997 infusion) in 11 patients with evaluable data. Post-treatment median *K*^trans^ values are in bold if more than five deciles in the corresponding histogram analysis of *K*^trans^ revealed statistically significant changes in the same direction as the change in the median value (see text and Figure 3).
